# Special issue “Photoacoustic imaging: microscopy, tomography, and their recent applications in biomedicine” in visual computation for industry, biomedicine, and art

**DOI:** 10.1186/s42492-021-00082-0

**Published:** 2021-05-31

**Authors:** Puxiang Lai, Liming Nie, Lidai Wang

**Affiliations:** 1 Department of Biomedical Engineering, The Hong Kong Polytechnic University, Hong Kong, SAR China; 2 Research Center of Medical Sciences, Guangdong Provincial People’s Hospital, Guangdong Academy of Medical Sciences, Guangzhou, 510080 China; 3 Department of Biomedical Engineering, The City University of Hong Kong, Hong Kong, SAR China

Photoacoustic (PA) imaging is a promising non-invasive and non-ionizing biomedical imaging modality that emerged in recent years. The articles presented in this special issue describe some of newest progress in this field. We are extremely grateful to all contributing authors.

The first part of the issue covers new laser source development, including fiber lasers and laser diodes. The second part is dedicated to improving the image resolution through chronic cranial window techniques, virtual-point concept, fast polygon scanning, and Fabry Perot sensing. The third part shows the basic principles of photoacoustic/ultrasound imaging and its applications.

## New laser source development

Jin and Liang describe recent developments in fiber-laser-based photoacoustic microscopy (PAM). Fiber lasers can be used to pump multiple wavelengths via different nonlinear effects. This approach is cost-effective and lightweight, and thus has been applied in handheld and head-mount PAM. Besides, fiber lasers have been used in all-fiber PA endoscopy for early cancer detection.

Li, Tsang, Kang, et al. present another cost-effective and compact light source, laser diodes (LD). Using a continuous-wave LD as the excitation source, they develop a high-speed high-resolution PAM system. A hybrid scanning mechanism that synchronizes a one-dimensional galvanometer mirror and a two-dimensional motorized stage is used for fast imaging. The PAM system has a high lateral resolution of 4.8 μm. *In vivo* microvasculature imaging is demonstrated with high signal-to-noise ratio.

## Improvement of image resolution

Wang and Xi describe a chronic cranial window technique as an effective tool for long-term *in vivo* imaging. Different chronic cranial windows have been fabricated to transmit more light through the skull in longitudinal imaging. The limitations and expectations for the PA cranial window are discussed in terms of lateral resolution, axial resolution, imaging depth, and stability.

Bai, Li, Ma, et al. introduce a virtual-point concept to fiber-sensor-based photoacoustic tomography to improve the image resolution. They demonstrate a fiber-laser virtual-point detector via bending the fiber into an arc. The curved fiber-laser sensor focuses on the arc center. A synthetic aperture focusing technique is used to significantly improve the elevational resolution beyond the focal zone. Via using a long cavity in the fiber laser sensor, a high numerical aperture and tight focus can be achieved to further increase the spatial resolution in the future. Compared with piezoelectric transducers, the fiber-laser detector is sensitive, immune to electromagnetism, lightweight, and flexible, making it attractive for high-resolution photoacoustic tomography.

Pan, Chen, Liu, et al. propose an image correction method based on accurate ultrasound positioning to improve the image quality of polygon-scanning photoacoustic microscopy. The photoacoustic and ultrasonic data are simultaneously acquired. The angle position of the polygon mirror is extracted from the ultrasonic data to correct the photoacoustic images. Results show an effective reduction of dislocations in the reconstructed image and much-improved image quality.

Yang, Zhang, Zeng, et al. describe a Fabry Perot sensor with improved signal-to-noise ratio and stability. Taking advantage of a double-cladding fiber, the sensing unit simultaneously couples the excitation and detection light for all-optical endoscopic detection. Furthermore, the microcavity-based sensor offers benefits of all-fiber integration, small size, and high sensitivity. The new miniaturized sensor can be used in non-destructive detection and biomedical applications.

## Multimodal imaging and its applications

Wang, Zhao and Xu describe in detail the basic principles of photoacoustic/ultrasound (PA/US) imaging and their applications. Their attention is given to the relationship between photoacoustic and ultrasonic imaging. Multi-modal and real-time imaging has become a focus and an important trend for PA technology. In particular, the combination of PA and ultrasonic imaging can not only be used for acoustic signal detection, but also provide more complementary information, improved accuracy, and thus better meet the needs in preclinical research and clinical diagnosis.

Chen and Tian describe the basic principles of PA technology for nondestructive testing and evaluation (NDT/E) and its applications. PAM, particularly OR-PAM, possesses a high spatial resolution to visualize fine structures and can achieve fast imaging speed. They show that the penetration depth of PA imaging can be extended via combining PA with US imaging. These technical advances can benefit NDT/E, structural health monitoring, and microscopy of metals.

In brief, the reported results in this issue demonstrate the rapid development of PA technology and the accelerated translation to clinical and other applications.

